# Social relations and health in an ethnically diverse social housing area selected for large structural changes compared to municipal levels: a Danish survey study

**DOI:** 10.1186/s12889-023-15034-x

**Published:** 2023-02-22

**Authors:** Monica F. Kvorning, Siv S. Nygaard, Abirami Srivarathan, Cathrine J. Lau, Rikke Lund

**Affiliations:** 1 Department of Public Health, Section of Social Medicine, University of Copenhagen, Copenhagen, Denmark; 2 Center for Clinical Research and Prevention, Bispebjerg and Frederiksberg Hospital, Capital Region of Denmark, Frederiksberg, Denmark; 3 Center for Healthy Aging, University of Copenhagen, Copenhagen, Denmark

**Keywords:** Social relations, Social support, Self-rated health, Neighborhood, Ethnicity

## Abstract

**Background:**

This study aims to describe demographics, social relations and health in an ethnically diverse social housing area selected to undergo large structural changes and compare it to the surrounding municipality. Furthermore, to explore the association between social relations and self-rated health (SRH) and the interaction with country of origin in both populations.

**Methods:**

Data sources include a multilingual interviewer-driven survey study in a social housing area (*N* = 209) and a municipal health survey (*N* = 1,638) among residents aged 45 + years. Information on social relations include contact frequency with and support from family, friends, and neighbors. Descriptive and multivariate logistic regression analyses adjusted for age, sex, and country of origin are presented, as well as joint effect analyses of social relations and country of origin on SRH.

**Results:**

In the social housing area, 38.8% of the respondents reported poor SRH compared to 19.5% in the municipality. In both study populations low contact frequency was associated with poor SRH, however insignificantly in the social housing area compared to the municipality sample, OR = 1.50 (0.65–3.46) vs. OR = 2.42 (1.70–3.45). Joint exposure to having non-Western background and low contact frequency was strongly associated with poor SRH in the social housing area, OR = 6.28 (1.80–21.89) but less so in the municipality, OR = 3.67 (1.55–8.69). The same tendency was seen regarding low support from social relations.

**Conclusions:**

This study provides insight to a population that is generally underrepresented in survey studies. In the social housing area, approximately twice as many reported poor SRH compared to the municipality data. In both populations, low contact frequency and low support were associated with poor SRH. Residents with weak social relations and non-Western origin simultaneously were more likely to report poor SRH in the social housing area specifically but less so in the municipality, indicating a higher vulnerability among the residents in the social housing area.

**Supplementary Information:**

The online version contains supplementary material available at 10.1186/s12889-023-15034-x.

## Background

Social relations are recognized as important social determinants of health [1]. Having poor social relations is known to increase the risk of mortality and morbidity [2–5]. Access to social support is suggested to buffer the health damaging effects of stressors [6]. Furthermore, several linking mechanisms have been suggested through health behaviors, mental wellbeing, and physiological factors [7]. Social relations are in most health research conceptualized into structural and functional aspects. The structural aspect involves quantitative measures such as frequency of contact, duration, and numbers of relations while the functional involves aspects of quality, including support, relational strain, and social anchorage [8].

Neighborhood characteristics can influence both social relations as well as health outcomes [9]. People living in more disadvantaged neighborhoods have been found to be at a higher risk of loneliness and social isolation [3]. Also, living in disadvantaged neighborhoods has been associated with poor self-rated health (SRH), heart disease and mortality [10–14]. Several mechanisms have been suggested, including walkability, access to healthy foods, tobacco availability, green spaces and levels of social connectedness and safety [15, 16].

Social housing areas are examples of often geographically well-defined spaces and thus an optimal arena for studying how changes may influence residents. In Denmark, close to 20% of the population live in non-profit social housing [17]. It comprises rental apartments and houses that are distributed based on waiting lists, but up to 25% may be assigned by the municipalities to individuals and families in specific need for housing due to, for example, divorce or to newly arrived refugees. The rent is fixed according to the costs and is generally affordable also with lower incomes and is in general of good quality [18]. A number of social housing areas, especially in and around the larger cities, have over time developed into areas with higher levels of socioeconomic deprivation and ethnic diversity. In an effort to improve these areas, many Western countries have issued alterations to the built environment [19, 20]. In Denmark, several social housing areas have been selected to undergo large structural changes in the coming years in order to change the composition of residents [21]. An implication of these changes is the rehousing of significant numbers of residents with potential negative consequences for established social relations and thus for health. Previous studies in Scandinavian social housing areas suggest that larger social networks, access to personal support and life resources is associated with better SRH [22–24], which is in line with population-based studies [25, 26]. SRH has been shown as a strong predictor for health. It includes the respondents’ own experiences and symptoms of both mental and physical health as well as functional ability due to both diagnoses given by health professionals as well as possible undiagnosed diseases because of its subjective character [27].

Residents in socio-economically disadvantaged areas and ethnic minorities are often underrepresented in survey studies [28, 29]. Perspectives from and experiences of these groups in general are thus largely missing in health and medical research [30].

Studies show that most ethnic minorities have a higher risk of poor SRH than the European natives [31]. Also, living in a deprived neighborhood in itself has been associated with an increased risk of disease and mortality[11, 14] and social relations have been suggested to buffer this stressful effect [32]. It is, however, to our knowledge unknown how social relations are associated with SRH in deprived ethnically diverse neighborhoods compared to the surrounding community, and if the vulnerability to poor social relations is larger among ethnic minorities, making them more susceptible to potential negative effects of large structural changes.

We hypothesize that strong social relations are associated with better SRH, and that residents in a deprived social housing area with non-Western background will be more vulnerable to exposure of poor social relations than those with Western background.

## Methods

The aim of the present study is to describe demographics, health status, socioeconomic characteristics, and social relations among middle-aged and older residents in a deprived ethnically diverse social housing area selected to undergo large structural changes and compare the results to municipal level. We further aim to study whether social relations are associated with SRH in the social housing area as well as in the municipality. Finally, we examine if residents with non-Western compared to Western origin are more vulnerable regarding the association between having poor social relations and poor SRH and compare results in the social housing area to the municipality.

This study is part of a larger multi-method research project, ‘Health, wellbeing and social relations in a changing neighborhood (Danish acronym: STRIT)’ [17]. It is based on survey data collected in 2018 in a social housing area located in a municipality outside of Copenhagen, Denmark. The social housing area was established in 1972 and consisted of a total of 917 apartments and approximately 2600 residents. The area is ethnically diverse with residents representing around 50 different nationalities. The survey was conducted during the rehousing of residents, prior to demolition of approximately a third of the social housing [29]. Residents aged 45 + years were included due to a previously identified need for health interventions among this group [33] and as these residents are often underrepresented in surveys [30]. Health, social relations and wellbeing were examined through a survey primarily employing validated instruments and items [29].

All residents over 45 years with legal residence in the housing area were retrieved from national registers through the unique Danish personal identification number, including data on sex, age, and country of origin.

The eight most prevalent languages were identified based on the residents’ country of origin, and the survey was translated from Danish to: Turkish, Arabic, Polish, Vietnamese, Urdu, Pashto, and English. In-person interviews were performed by multilingual interviewers from September to December 2018. Residents were contacted until they either accepted or rejected participation or the data collection period ended. The mean number of contact attempts was 4 [29].

Prior to the survey data collection, the research group attended neighborhood events and used posters, invitation letters and social media to raise awareness about the survey. Cooperation with the resident ambassadors and the social housing administration was initiated to facilitate recruitment of respondents.

The results from the survey in the social housing area were compared to a survey conducted in the municipality where the social housing area is located, called The Danish Capital Region Health Survey (DK-CRHS). This survey was conducted using a mixed-mode approach, allowing individuals to complete a web questionnaire or a paper questionnaire from February to May 2017 and was part of The Danish National Health Survey [34]. The DK-CRHS included randomly selected residents from the municipality and was available in Danish. The survey contained questions similar to those of the STRIT survey. The municipality had a lower level of education, employment rate and income and a higher share of non-Western residents compared to other municipalities in the region surrounding and including Copenhagen [35].

### Sample and study respondents

The study sample for STRIT consisted of 604 residents of which 209 participated (35% response rate). Non-Western residents constituted 66.7% of the original study sample and 67.0% of the respondents.

The DK-CRHS municipality study sample consisted of 4,500 randomly selected adults from the entire municipality with a response rate of 51.7% (*N* = 2,532). This study includes 1,638 residents aged 45 + years with a response rate of 62.0%. In the original DK-CRHS study sample of the municipality, 12.5% of the residents had non-Western origin but amounted 7.1% of the respondents.

Full information on all covariates were requested for the multivariate analyses.

### Primary measures

#### Social relations, structural aspect

In STRIT, the structural aspect of social relations was assessed by cohabitation status and frequency of contact employing the validated Copenhagen Social Relations Questionnaire including the questions: ‘How often are you together with any of the following persons, who you do not live with?’ and ‘How often do you have contact with the following persons, without seeing them? (E.g. by telephone, Skype, letter, email, text message, Messenger, WhatsApp, Viber etc.)’ referring to six roles: partner, parents, children, other family, friends, and neighbors or other residents in the area [36].

In the DK-CRHS, the question regarding contact frequency was slightly different, asking “How often are you in contact with friends, acquaintances, and family that you do not live with? (Contact refers to physically being together, talking on the telephone together, writing each other etc.)” referring to the roles: family, friends, colleagues and fellow students in your spare time, neighbors and people from your neighborhood, and people you have met on the internet. Items representing the roles family, friends and neighbors were included in this study for comparability.

In both STRIT and DK-CRHS, contact frequency was dichotomized into ‘often’ when cohabitating or having contact at least once a month, and ‘seldom’ when contact less than once a month.

#### Social relations, functional aspect

In STRIT, support was assessed by asking “Can you talk with any of the following people, if you need support?” referring to the same six roles. Response options were: “Always”, “Often”, “Sometimes”, “Seldom”, “Never”, or “Not relevant”. If respondents replied “Never” or “Not relevant” support was categorized as low and else as high.

The DK-CRHS assessed support by asking “Do you have somebody to talk to if you have problems or need support?” with the response categories: “Yes, always”, “Yes, often”, “Yes, sometimes” and “No, never or almost never”. If respondents replied “No, never or almost never” support was categorized as low, and else as high.

**Fig. 1:**
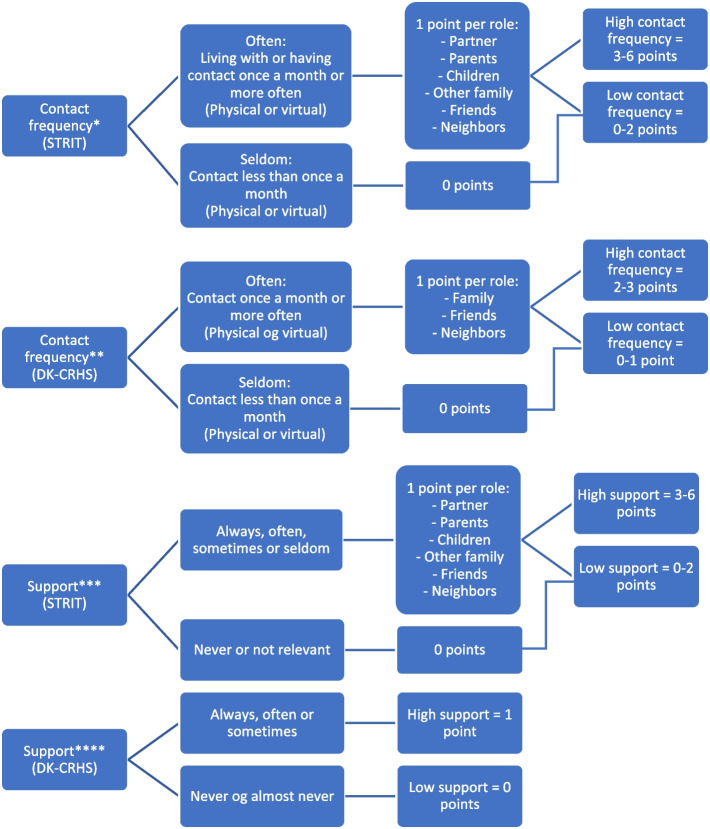
Operationalization of constructed measures of contact frequency and social support. * Living with someone or having contact at least once a month counted 1 point pr. role, range 0–6. Having 0–2 points was categorized as low contact frequency and 3–6 was categorized as high. ** Living with someone or having contact at least once a month counted 1 point pr. role, range 0–3. Having 0–1 point was categorized as low and 2–3 as high contact frequency. *** Having access to support always, often, sometimes, or seldom counted one point pr. role, range 0–6. Having 0–2 points was categorized as low support and 3–6 as high. **** Having access to support from someone always, often, or sometimes was categorized as high support and having access never or almost never as low support

Based on the dichotomization, a social relations index was constructed to divide contact frequency and support into high vs. low depending on the number of social roles. The social relations index is illustrated in Fig. 1.

Two joint variables between contact frequency and support, respectively, and Western/non-Western origin were constructed, creating eight groups, four regarding contact frequency and four regarding support: Western with high contact frequency/support (reference groups), Western with low contact frequency/support, Non-Western with high contact frequency/support, and Non-Western with low contact frequency/support.

#### Health

Short-Form 12/36 was used to assess SRH by asking “In general, would you say your health is excellent, very good, good, fair or poor?” [28, 36], dichotomized into good (excellent/very good/good) or poor (fair/poor) in both STRIT and the DK-CRHS. The other measure of health assessed was the number of current chronic diseases based on a predefined list in the survey (maximum of 18).

#### Socioeconomic characteristics

In both STRIT and DK-CRHS, information was derived from questionnaire. Employment status was divided into currently employed or not. Educational level was divided into higher than ninth grade and ninth grade or less.

#### Country of origin

Country of origin was divided into Western (31 EEA countries and Andorra, Monaco, San Marino, Switzerland, Vatican State, Canada, USA, Australia, and New Zealand) and Non-Western (all other countries). If the residents in STRIT had Western origin based on register data but had reported non-Western origin in the survey, they were categorized as non-Western. In DK-CRHS, country of origin was based on register data only.

### Analyses

Descriptive analyses of the selected variables and multiple logistic regression analyses were conducted using the SAS Enterprise guide 7.1 software. Multiple logistic regression analyses were performed to assess: 1) the association between social relations and SRH in analyses adjusted for sex, age, and country of origin and 2) the joint effect of social relations and country of origin on self-rated health in analyses adjusted for sex and age. Descriptive results are presented as numbers and percentages, and the multiple logistic regression analyses results as odds ratios (OR) with 95% confidence intervals (CI).

## Results

The distribution of main characteristics of the two study populations are presented in Table 1. In STRIT, 38.8% of the respondents reported poor SRH compared to 19.5% in DK-CRHS. STRIT respondents reported 3.1 chronic illnesses per person on average compared to 1.7 in the municipality sample. In both populations, there was an almost equal distribution in sex but the DK-CRHS had a higher representation among older age groups than the STRIT population. The proportion of low educated and unemployed residents was considerably higher in STRIT compared to the municipal sample (55.3% vs. 29.7% and 68.3% vs. 51.0%) and even more pronounced within the group of non-Western residents. Among these, 69.8% had low educational level and 73.4% were unemployed in STRIT while in DK-CRHS, only 23.8% had low educational level and 50.9% were unemployed (results not shown). The proportion of residents with low contact frequency as well as low social support was also considerably higher in STRIT compared to the municipal sample (18.5% vs. 10.1% and 19.1% vs. 5.4%). In STRIT, 56.8% had high contact frequency with their neighbors or other residents in the area and 58.9% reported to have high access to support from them. In DK-CRHS, 73,5% had high contact frequency with their neighbors. Questions regarding support from neighbors specifically was not included in the DK-CRHS.

**Table 1:** Distribution (N (%)) of sociodemographic variables and measures of social relations in the two cohorts

	**STRIT**	**DK-CRHS**
N (%)	209 (100.0)	*1,638 (100.0)*
**Sex**		
Male	103 (49.3)	825 (50.4)
Female	106 (50.7)	813 (49.6)
**Age**		
45–49 years	44 (21.1)	233 (14.2)
50–69 years	119 (56.9)	918 (56.1)
70 + years	46 (22.0)	487 (29.7)
**Country of origin**		
Western	69 (33.0)	1521 (92.9)
Non-western	140 (67.0)	117 (7.1)
**Self-rated health**		
Good	126 (61.2)	1310 (80.5)
Poor	80 (38.8)	318 (19.5)
**Educational level**		
More than 9^th^ grade	92 (44.4)	1108 (70.3)
9^th^ grade or less	115 (55.6)	469 (29.7)
**Employment status**		
Currently employed	66 (32.0)	782 (49.0)
Unemployed	140 (68.0)	815 (51.0)
**Contact frequency**		
High	168 (81.5)	1472 (89.9)
Low	38 (18.5)	166 (10.1)
**Support**		
High	165 (80.9)	1517 (94.6)
Low	39 (19.1)	87 (5.4)
**Contact frequency with neighbors or other residents in the area**		
High	117 (56.8)	1167 (73.5)
Low	89 (43.2)	421 (26.5)
Support from neighbors or other residents in the area^*^		
High	119 (58.9)	-
Low	83 (41.1)	-
**Average no. of current chronic illnesses**	3.1	1.7

STRIT data collected in 2018 in the social housing area and the DK-CRHS data collected in 2017 in the municipality

^*^The DK-CRHS does not contain a question regarding access to support from neighbors

Multivariate logistic regression analyses of the association between the social relation variables and SRH are presented in Table 2. In both study populations low contact frequency was associated with poor SRH, however insignificantly in STRIT, OR = 1.50 (0.65–3.46) vs. OR = 2.42 (1.70–3.45) in the municipality. The association between low support and poor SRH points in the same direction, (OR = 1.63 (0.74–3.63)) in STRIT and OR = 3.05 (1.93–4.83) in DK-CRHS.

**Table 2:** Multiple logistic regression analyses on the association between contact frequency/support and self-rated health (SRH)

	**Poor SRH in STRIT**	**Poor SRH in DK-CRHS**
	OR (95% CI)	OR (95% CI)
*N (%)*	*209 (100.0)*	*1,638 (100.0)*
**Contact frequency**	*N* = 205	*N* = 1,628
High	1.00 (ref.)	1.00 (ref.)
Low	1.50 (0.65–3.46)	2.42 (1.70–3.45)
**Support**	*N* = 203	*N* = 1,596
High	1.00 (ref.)	1.00 (ref.)
Low	1.63 (0.74–3.63)	3.05 (1.93–4.83)

All analyses are adjusted for age, sex, and Western/non-Western origin

*OR* odds ratio, *CI* confidence intervals

**Fig. 2:**
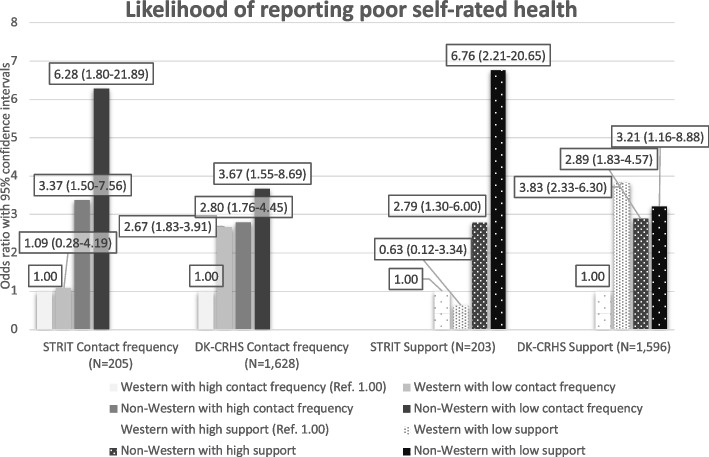
Joint effect of measures of social relations and country of origin on self-rated health (SRH)

Figure 2 shows the analyses between the joint variables of country of origin and measures of contact frequency and support, respectively, in relation to SRH. In STRIT, individuals with non-Western origin and low contact frequency or low support were most likely to report poor SRH compared to individuals with Western origin with high contact frequency or high support. Results indicate a synergistic effect when having both non-Western origin and low contact frequency/support. The odds ratios for these groups were higher than the combined odds ratios of the groups of Western residents with low contact frequency/support and non-Western residents with high contact frequency/support. This synergistic effect was not observed to the same extent regarding contact frequency and not at all regarding support in the municipal sample.

### Sensitivity analyses

Adjusting for educational level and employment status did not alter the results of neither of the analyses significantly, see Additional file 1.

## Discussion

The descriptive results of the present study indicate a pronounced higher morbidity, poorer social relations and lower socioeconomic position in the social housing area compared to the municipality it is located in. The participants of the present study only include residents aged 45 + years. A higher morbidity is therefore to be expected in comparison to other survey studies that include participants of younger ages as well.

In line with previous findings in population-based studies [22, 23], the current study suggests an association between poor social relations, measured by contact frequency and support, and poor SRH. This is applicable to both study samples, however non-significant in STRIT. The difference in the strength of the association between social relations and SRH between the two populations is most likely due to the smaller population in STRIT. However, the different characteristics of the two populations regarding socioeconomic status, country of origin, and health, as well as social relations may also influence the differences in estimates e.g. through differences in residual confounding. Despite the lack of a formally significant association in the STRIT population, groups within the area who are specifically vulnerable to exposure to poor social relations exists, as demonstrated in the present study. Previous literature has found that living in a deprived neighborhood, having limited social network and being part of an ethnic minority are all factors associated with poor SRH [14, 22, 31]. The results of the present study further indicate that double exposure to being of non-Western background and having poor social relations is associated with poor SRH when also living in a deprived social housing area. This was not seen in the municipality sample to the same extent. Having non-Western background therefore seems to increase the vulnerability of having poor social relations when also living in an ethnically diverse deprived social housing area but not in the background population. This could be due to differences in social position between the STRIT and DK-CRHS populations. However, sensitivity analysis with adjustment for educational level and employment status did not alter the results significantly, but residual confounding from unmeasured socioeconomic circumstances or other factors cannot be excluded. Another explaining factor could be the limited proportion of non-Western residents in DK-CRHS sample. The DK-CRHS was only available in Danish, which can increase the risk of non-response among ethnic minorities[28] that are known to have a higher risk of poor health [31].

In STRIT, the representation of non-Western residents was equal to the one in the original sample population (see Additional file 2). In contrast, in DK-CRHS, non-Western residents constituted a smaller percentage in the study population compared to the original sample population and was as such not representative of the population in the municipality. This may introduce selection bias to our analyses and the true difference between for example the joint effects analyses in the two populations may be smaller than suggested by the present results.

In the social housing area, more than half reported to have high contact frequency with and support from their neighbors. When a third of the social housing within the area will be demolished and the residents rehoused, social ties between residents are likely to be affected negatively and, in turn, their health [37, 38]. The results of this study point towards the possibility that residents within the area will be affected additionally by loss of social relations. Quantitative studies on how demolition and rehousing affect social relations within a neighborhood is to our knowledge missing. What is known, however, are some of the different neighborhood factors that positively influence health, including increased access to green spaces, formal and informal social networks, healthcare services, and participation in recreational activities [9, 14, 39]. An awareness on these as well as efforts to preserve and create social ties are potential ways to buffer residents’ health following the large structural changes.

It is important to give voice to the residents who will be directly affected by the large structural changes in order to follow the consequences of these over time and perhaps consider preventive social interventions [22, 32, 40]. Although this study is specific to the context of the researched area, there are many other social housing areas in Denmark with similar characteristics, and the same associations between social relations, country of origin and SRH are likely to be found there as well. These areas will undergo similar large structural changes in the coming years.

## Strengths and limitations

Ethnic minorities and socially disadvantaged groups are often underrepresented in health research [30]. The strength of this study is therefore the achievement of reaching these, especially the non-Western residents, by using a translated survey performed face-to-face by a multilingual interview corps, making several contact attempts at residents’ addresses and by creating trust through local ambassadors. Another strength is the availability of comparable data from the surrounding municipality making it possible to study the effects of living in a deprived social housing area vs. the more general population. However, it is a limitation of the study that not exactly comparable variables were available for the measures of social relations. Of note, the outcome measure was identical, and careful coding of the exposure measures into indices of social relations allowed for a good similarity between the selected items. It cannot be ruled out that differences in the associations could be ascribed to differences in wordings, however the item formulations were quite close regarding both the structural and functional aspect of social relations.

The response rate in STRIT was 35% and relatively low which led to broad confidence intervals in all analyses as well as an increased risk of selection bias. This was despite the numerous efforts to reach as many residents as possible. Study respondents are often more resourceful than non-respondents and the same can be assumed for the STRIT sample [30]. However, the sample of 209 respondents share similar demographic characteristics as the non-respondents in the housing area, which limits the risk of selection bias (see Additional file 2). Further details regarding data collection are discussed elsewhere [29]. The qualitative sub-studies in the STRIT project performed in the area has provided an insight into the reasons for non-participation in the surveys. Srivarathan et al. and Nygaard et al. found that residents expressed ‘Participation fatigue’ due to the many projects in the housing area performed by several research teams, among these STRIT, as well as the municipality. Also, residents expressed a lack of motivation to participate actively in the area when they might have to move due to the upcoming demolitions [29, 41].

## Conclusions

This study provides insight to a population that is known for being underrepresented in population studies. The present study found a higher morbidity, poorer social relations and lower socioeconomic position in the social housing area compared to the municipality it is located in. Poor social relations were associated with poor SRH in the municipality cohort with a tendency in the same direction for the smaller STRIT population. However, when having both non-Western origin and poor social relations, the association with having poor SRH was much more profound within the social housing area. This indicates a higher vulnerability towards having poor social relations among residents with non-Western background in the social housing area. The planned large structural changes could potentially increase the risk for residents losing social relations, and therefore points toward the need for preventive initiatives to preserve and develop new social ties within the area as well as a larger awareness of the potential health damaging effects of a weakened access to social support during the large structural changes.

## Supplementary Information

**Additional file 1.** Sensitivity analyses of multivariate logistic regression analyses when adjusting for socio-economic factors.

**Additional file 2.** Characteristics of respondents and the original sample population in STRIT.

## Data Availability

Access to data can be requested by contacting Rikke Lund (rilu@sund.ku.dk).

## Abbreviations

SRH: Self-rated health
STRIT: The survey named “Health, wellbeing and social relations in a changing neighborhood”
DK-CRHS: The Danish Capital Region Health Survey
OR: Odds ratio
CI: Confidence intervals
