# Supplementary material for: Social relations and health in an ethnically diverse social housing area selected for large structural changes compared to municipal levels: a Danish survey study
Source: BMC Public Health. 2023 Feb 22;23:379. doi: 10.1186/s12889-023-15034-x (PMC9948324; doi:10.1186/s12889-023-15034-x)
Supplement: Supplementary file 1 — Additional file 1. Sensitivity analyses of multivariate logistic regression analyses when adjusting for socio-economic factors. [file 12889_2023_15034_MOESM1_ESM.docx]

***Additional file 1.*** *Sensitivity analyses of multivariate logistic regression analyses when adjusting for socio-economic factors.*

|  | STRIT OR (95% CI) | STRIT* OR (95% CI) | STRIT** OR (95% CI) | DK-CRHS OR (95% CI) | DK-CRHS* OR (95% CI) | DK-CRHS** OR (95% CI) |
| --- | --- | --- | --- | --- | --- | --- |
|  | Poor SRH | | | | | |
| Cross-sectional analyses | | | | | | |
| Contact frequency | (N=205) | (N=205) | (N=205) | (N=1,628) | (N=1,570) | (N=1,591) |
| High | 1.00 (ref.) | 1.00 (ref.) | 1.00 (ref.) | 1.00 (ref.) | 1.00 (ref.) | 1.00 (ref.) |
| Low | 1.50 (0.65-3.46) | 1.44 (0.63-3.33) | 1.73 (0.73-4.11) | 2.42 (1.70-3.45) | 3.03 (2.08-4.42) | 2.82 (1.92-4.14) |
| Support | (N=203) | (N=203) | (N=203) | (N=1,638) | (N=1,564) | (N=1,638) |
| High | 1.00 (ref.) | 1.00 (ref.) | 1.00 (ref.) | 1.00 (ref.) | 1.00 (ref.) | 1.00 (ref.) |
| Low | 1.63 (0.74-3.63) | 1.66 (0.74-3.69) | 1.42 (0.63-3.16) | 3.05 (1.93-4.83) | 3.25 (2.04-5.19) | 3.15 (1.96-5.06) |
| Cross-sectional analyses with joint variables | | | | | | |
| Contact frequency | (N=205) | (N=205) | (N=205) | (N=1,628) | (N=1,570) | (N=1,591) |
| Western and high contact frequency | 1.00 (ref.) | 1.00 (ref.) | 1.00 (ref.) | 1.00 (ref.) | 1.00 (ref.) | 1.00 (ref.) |
| Western and low contact frequency | 1.09 (0.28-4.19) | 1.09 (0.28-4.22) | 1.07 (0.27-4.24) | 2.67 (1.83-3.91) | 3.36 (2.25-5.01) | 3.24 (2.15-4.87) |
| Non-Western and high contact frequency | 3.37 (1.50-7.56) | 4.05 (1.67-9.80) | 2.67 (1.16-6.13) | 2.80 (1.76-4.45) | 2.66 (1.63-4.35) | 2.17 (1.33-3.52) |
| Non-Western and low contact frequency | 6.28 (1.80-21.89) | 7.05 (1.99-24.95) | 6.52 (1.76-24.12) | 3.67 (1.55-8.69) | 4.19 (1.64-10.69) | 2.65 (1.03-6.84) |
| Support | (N=203) | (N=203) | (N=203) | (N=1,628) | (N=1,564) | (N=1,582) |
| Western and high support | 1.00 (ref.) | 1.00 (ref.) | 1.00 (ref.) | 1.00 (ref.) | 1.00 (ref.) | 1.00 (ref.) |
| Western and low support | 0.63 (0.12-3.34) | 0.65 (0.12-3.45) | 0.47 (0.09-2.52) | 3.83 (2.33-6.30) | 3.99 (2.41-6.63) | 4.16 (2.49-6.95) |
| Non-Western and high support | 2.79 (1.30-6.00) | 3.30 (1.42-7.65) | 2.18 (0.98-4.87) | 2.89 (1.83-4.57) | 2.72 (1.68-4.39) | 2.29 (1.42-3.69) |
| Non-Western and low support | 6.76 (2.21-20.65) | 8.00 (2.48-25.78) | 4.96 (1.58-15.56) | 3.21 (1.16-8.88) | 3.42 (1.21-9.70) | 2.06 (0.71-5.96) |

*All analyses are adjusted for age, sex and Western/non-Western origin.
OR: odds ratio; CI: confidence intervals.
*Adjusted for educational level (Above 9^th^ grade (ref.) or 9^th^ grade or less).
**Adjusted for employment status (currently employed (ref.) or not).*
