# Supplementary material for: Social relations and health in an ethnically diverse social housing area selected for large structural changes compared to municipal levels: a Danish survey study
Source: BMC Public Health. 2023 Feb 22;23:379. doi: 10.1186/s12889-023-15034-x (PMC9948324; doi:10.1186/s12889-023-15034-x)
Supplement: Supplementary file 2 — Additional file 2. Characteristics of respondents and the original sample population in STRIT. [file 12889_2023_15034_MOESM2_ESM.docx]

**Additional file 2*.*** *Characteristics of respondents and the original sample population in STRIT.*

|  | STRIT respondents | STRIT Original sample population |
| --- | --- | --- |
| N (%) | 209 (100) | 604 (100) |
| Sex |  |  |
| Male | 103 (49.3) | 322 (53.3) |
| Female | 106 (50.7) | 282 (46.7) |
| Age |  |  |
| 45-49 | 44 (21.1) | 147 (24.3) |
| 50-69 | 119 (56.9) | 354 (58.6) |
| 70+ | 46 (22.0) | 103 (17.1) |
| Origin |  |  |
| Western | 69 (33.0) | 201 (33.3) |
| Non-Western | 140 (67.0) | 403 (66.7) |
